# Adoption of harmonisation policy for the midwives’ training programme in Mali: A policy analysis

**DOI:** 10.1371/journal.pgph.0001296

**Published:** 2022-11-29

**Authors:** Cheick S. Sidibé, Valentine Becquet, Tanya Y. Brückner, Ousmane Touré, Lalla Fatouma Traoré, Jacqueline E. W. Broerse, Marjolein Dieleman

**Affiliations:** 1 Institut National de Formation en Sciences de la Santé, Bamako, Mali; 2 Athena Institute for Research on Innovation and Communication in Health and Life Sciences, Vrije Universiteit, Amsterdam, Netherlands; 3 Institut national d’études démographiques (Ined), Aubervilliers, France; 4 Département d’Enseignement et de Recherche en Santé Publique, Faculté de Médecine et d’Odontostomatologie, Bamako, Mali; University of Regina, CANADA

## Abstract

West Africa is engaged in a process of harmonising health workers’ training programmes as a means to regulate regional training standards and thus improve their quality. There is currently a lack of documented information regarding the adoption of these revised training programmes. In 2012 a harmonised programme, the WAHO competency-based curriculum, was introduced in Mali for training midwives. The present study explores the barriers and facilitators of the adoption of this programme and how the content, context, process, and actor-related factors influenced this. We used a qualitative research design consisting of document analysis (n = 25) and semi-structured interviews (n = 21) with policymakers, students, and those in charge of implementing the training programme. Information was collected on education and training policies, the context and process of the harmonised curriculum development, its adoption, and the actors involved in the adoption strategy, along with their role. The study shows that the adoption of the harmonised curriculum in Mali offered midwives an opportunity to attain a higher standard of training and level of qualification than before. It also displayed both the government’s and the public school’s willingness and commitment to improve maternal and child health through enhancing midwives’ training standards. The most salient factors that influenced adoption were the lack of available resources, and the lack of involvement of, and coordination with, relevant actors for successful policy adoption. Mali’s experience of adopting the harmonisation policy of training curricula demonstrates the need for the authorities to collaborate with relevant actors for information dissemination and in the adoption process. It also demonstrates the need for finding innovative ways to secure and diversify funding opportunities, as well as establish a supervisory body for health worker training.

## Introduction

The global maternal mortality ratio (MMR) was estimated at 211 per 100,000 live births in 2017, reduced by 38% since 2000 [1, 2]. Despite this progress, maternal mortality remains a major public health concern in low- and middle-income countries (LMICs), particularly in sub-Saharan Africa, which accounts for approximately two-thirds of maternal deaths worldwide [3]. The most effective strategies to reduce the MMR include access to improved ante- and post-natal care, access and ability to choose adapted contraceptive methods, safe abortion, and to increase births attended by skilled health personnel [4–6]. In this paper, “skilled health personnel” refers to midwives (“sage-femme”) and does not include matrons (community midwives) or obstetric nurses, who also attend a large number of births in Mali.

Globally, the introduction of educated, trained, and motivated midwives has been associated both with a rapid and sustained decline in maternal and new-born mortality, and with an improvement in quality of care [7]. Therefore, improving training to produce midwives with necessary competencies is an essential strategy for improving maternal and child health [8–11].

In 2018, Mali’s MMR was estimated at 325 deaths per 100,000 live births [12]. This is substantially higher than the United Nations Sustainable Development Goal (SDG) 3, which aims to reduce the global MMR to below 70 per 100,000 live births by 2030 [3]. In 2018, 67% of births in Mali occurred in a health facility, of which only 46% were attended by any skilled health personnel [12]. These statistics exemplify the need for increased births attended by skilled health personnel in Mali.

In 1998 the heads of the Economic Community of West African States (ECOWAS) established the West African Health Organisation (WAHO), which encompasses 15 countries in the region, including Mali. Its vision is to promote better health through regional integration–specifically, through increased coordination, collaboration, and cooperation. The WAHO has highlighted improved pre-service education at the heart of its strategic plan, with a focus on harmonising the curricula, accreditation criteria, standards of practice, and codes of ethics for nurses and midwives across the region [13–16]. Its approach to harmonisation is in line with regional needs, based on a regionally accepted, competency-based curriculum in reproductive, maternal, new-born, and child health. It focuses on ensuring minimum standards for entry into midwifery practice. The curriculum was developed in workshops involving representatives of regional associations and councils, midwives, nurses, and managers of training institutions from the WAHO’s member states–including Mali’s public training institute for midwives and nurses, the National Training Institute in Health Sciences (INFSS). In 2012, the curriculum was validated and adopted by member states. The harmonised programme provides access to a bachelor’s degree in midwifery, thus resolving the disparity of designations and qualifications of midwives trained in different regional and national institutions [17].

In addition to Mali’s adoption of the WAHO’s harmonised curriculum, efforts to improve maternal and new-born survival and health included the expansion of health facilities’ networks and improvements in the quality of care. Midwives are key frontline health professionals for delivering quality care to pregnant and labouring women and new-borns in primary health care facilities. They provide perinatal care, manage normal deliveries, and detect and refer complications. To be certified, they complete an approved training programme and pass a national certification exam.

Midwives’ training in Mali has evolved considerably over the past 50 years. From 1963 to 1998, midwives were trained to the level of health technicians, where they underwent three years of training after nine years of education. In 1995, midwives’ training, previously provided by the public schools, was opened to the private sector. Access to the training was made conditional on the baccalaureate diploma (12 years of basic training) in 1998, and midwives became senior health technicians. This meant they had three years’ training after the baccalaureate but not a bachelor’s degree, since Mali had not yet moved to the Bachelor (Licence)-Master-Doctorate (LMD) system.

The midwife educational programme has undergone several changes since 2003, when the curriculum was updated to include emergency obstetric and neonatal care. This version, an objective-based curriculum–commonly referred to as the “classic curriculum”–was used in all training schools. In 2004, the INFSS was created by merging the existing public schools. It introduced and implemented a competency-based curriculum to midwifery training in Mali in 2008. This curriculum was more oriented towards the acquisition of practical skills adapted to the context compared to the objective based curriculum. However, private schools did not adopt the competency-based curriculum.

Both curricula remained in place until 2012, when the WAHO harmonised curriculum for midwives was introduced in Mali as part of a policy to improve the quality of training on maternal and child health. The WAHO curriculum was adopted by the INFSS in 2012, replacing the 2008 competency-based curriculum. However, it has not fully been introduced in the 30 private schools that currently train most midwives in Mali. In 2020, 142 midwives graduated from the INFSS under this new curriculum, and 574 midwives graduated from private schools under the objective-based curriculum [18]. Put into perspective, this means that just under 20 percent of all newly trained midwives in 2020 were trained with the WAHO curriculum. It is likely that graduates from private schools, who are the majority of newly graduated midwives, have different skills and qualifications due to different training curricula from those graduating from the public school when they enter the labour market.

While existing literature focuses largely on analysing policy implementation, limited attention is paid to other aspects of the policy process, such as policy adoption [19]. To the best of our knowledge, there is no literature on the policy adoption process in Mali. Although the WAHO harmonised curriculum was recommended for training in 2012, there are no research reports on its in-country adoption and implementation. It is critical to consider which factors influence policy adoption, and to describe and discuss adoption strategies, stakeholders’ involvement, and the influence of contextual factors to better understand why, or why not, a policy was adopted [20]. In this study, we aimed to explore barriers and facilitators in the actual adoption of the harmonisation policy for midwifery training in Mali and how the content, context, process, and actor-related factors influenced this. An understanding of the factors associated with the curriculum adoption can help identify receptive contexts in which to launch a new training initiative. This study can also inform plans to change existing training policies and the development of future policies. In this study, policy adoption refers to the change of curriculum to the harmonised WAHO competency-based curriculum for pre-service training.

## Materials and methods

**Fig 1:**
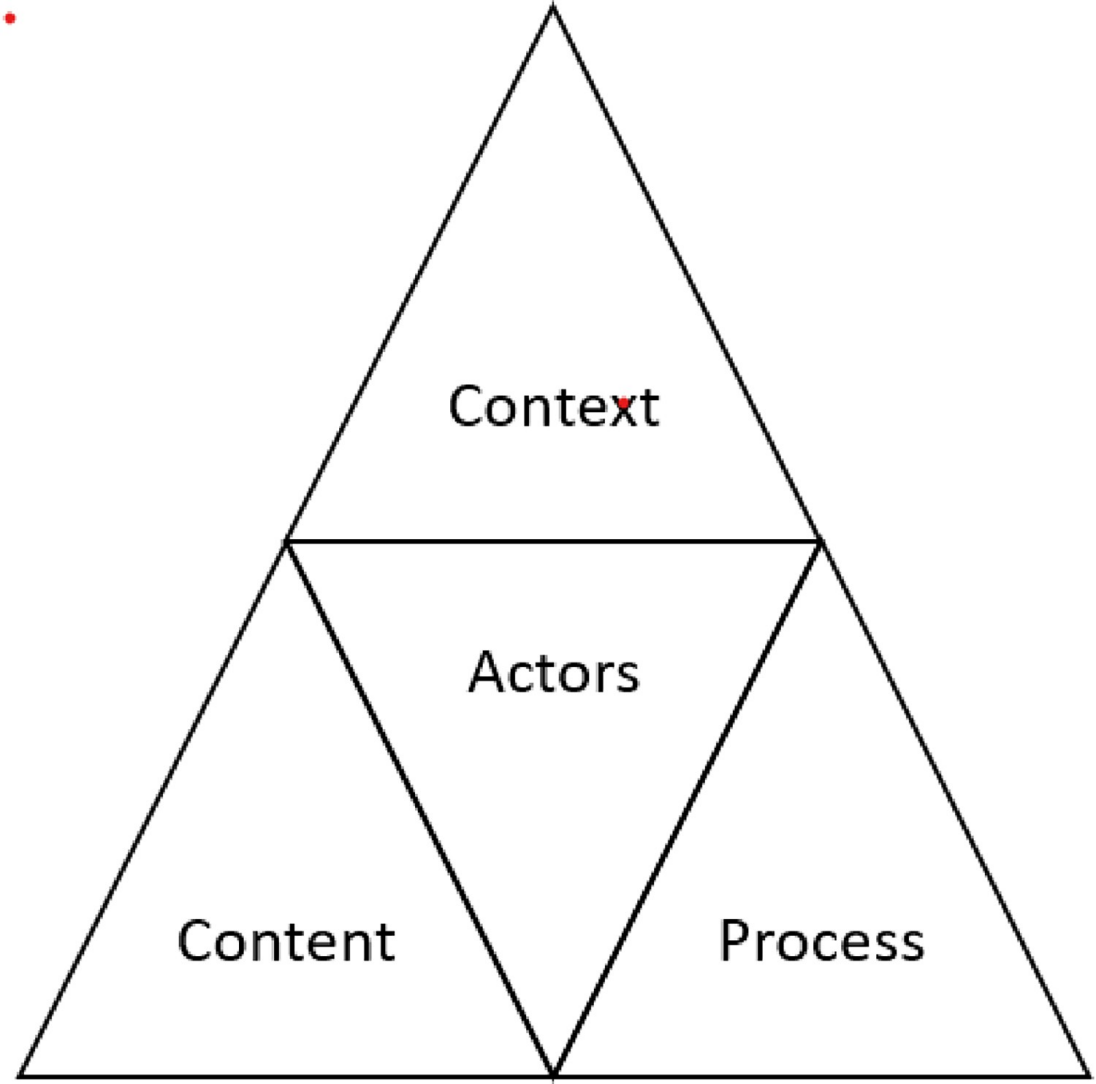
Policy analysis triangle from Walt and Gilson [19].

This study used qualitative research through document analysis and semi-structured interviews. The study approach was informed by Walt and Gilson’s policy analysis triangle [19], which identifies four interrelated components (policy content, actors, process and context,) that influence policy development, adoption and implementation (Fig 1). ‘Context’ refers to political, economic, and social factors at a national and international level, while ‘process’ refers to the way policies are initiated, formulated, implemented, and evaluated. ‘Policy content’ refers to the substance of a particular policy. ‘Actors’ are individuals and organisations that influence these different stages in policy making and the policy content. The framework describes the interactions between the four components. The conceptual framework has been used for policy analysis in different countries and contexts, including LMICs [21–25]. We used this framework to inform data collection and analysis of the adoption of the WAHO’s harmonisation policy on the midwifery training curriculum. We examined the policy objectives and content, the political, legislative, and social context in which the curriculum has been adopted, the process of the policy dissemination and adoption, and the actors involved in the process.

### Data collection

Data were collected in 2018 by two trained researchers from the department of public health at the University of Sciences, Technics and Technologies of Bamako and by the first and the second authors.

### Document review

We systematically collected and reviewed education and training policies, laws and directives issued by international organisations with which Mali is affiliated, such as the ECOWAS, WAHO, the West African Economic and Monetary Union, and those of national legislative bodies such as the Ministry of Higher Education (MoHE), Ministry of Health (MoH), and Ministry of Public Service. We also collected and reviewed documents issued by or related to the INFSS and private training schools, including documents related to the establishment of the institution, the organisation of the training programme, the functioning of the structure, accreditation and programme approval documents, training programmes, internal regulations, activity reports, and reports on adoption and implementation of the WAHO’s harmonised programme.

We collected and reviewed 25 documents based on the authors’ knowledge of the programme, recommendations from interviewees, follow-up of references within documents, and a request for information made to the training schools and to the Ministerial Departments of Health and Education. Each document was scanned through for relevance, read in detail, and included after being deemed to be of potential significance. The inclusion criteria were documents presenting information on midwives’ training and regulation in Mali. We started by identifying and analysing directives and laws on education, including in midwifery, issued by sub-regional organisations to which Mali is affiliated and those of national legislative bodies, and then examined how directives were implemented.

### Semi-structured interviews

To examine the experiences and perceptions of stakeholders on the adoption of the WAHO competency-based curriculum, as well as to gain deeper insight into contextual factors influencing adoption, we conducted semi-structured face-to-face interviews with individuals from organisations who are directly involved in the development or adoption of the curriculum or are influenced by changes in curriculum. A purposeful sampling strategy was used to select, and recruit participants based on their involvement in the harmonisation policy process or being a stakeholder in the adoption process [26].

We identified informants in discussions with respondents from training schools’ and a former senior official of the MoH. Subsequently, we used snowball sampling to further identify potential respondents. The participants in the study consisted of a diverse group of stakeholders that would be influenced by or have an interest in changes in curriculum for midwives, ranging from policy makers and training schools, to students, midwives, and employers (see Table 1 for details). In total, we conducted 21 interviews at the workplaces of the different participants. Interviews were conducted in French and lasted approximately 45 minutes. We used a pretested interview guide structured around the four components of the policy analysis triangle. All interviews were audio recorded after informed consent was obtained from participants. No remuneration was provided. Table 1 details the participants’ affiliations.

**Table 1:** List of study participants (n = 21).

1. Government organisations (n = 5)
• Representative of National Directorate of Human Resources for Health
• Representatives of General Directorate of Health (n = 2)
• Representative of General Directorate of Higher Education
• Representative of National directorate of communities Public Services
2. Training schools (n = 5)
• Representatives of public training schools (n = 3)
• Representatives of two private training schools (n = 2)
3. Health structures (n = 2)
• Employers of public health facilities
4. Graduates (n = 4)
• Midwives graduated in the new programme
5. Associations and professional councils (n = 2)
• Representative of midwives’ association
• Representative of midwives’ association and council
6. Technical and Financial partners (TFP) (n = 3)
• Representative of the Sahel Women’s Empowerment and Demographics Project (SWEDD)
• Representative from UNFPA
• Intra Health Capacity Plus Programme

### Data analysis

Data were analysed using NVivo qualitative software version 12. Interview records were transcribed verbatim and thereafter translated into English by the primary researcher, who speaks fluent French and English. Data was coded deductively using a coding frame, and inductively, by adding codes emerging from the data. In more detail: The first author used the main components of the Policy Triangle to develop a coding frame with four core-themes: content, actors, process and context. For each theme, codes were developed for subthemes. The usefulness of these codes was tested by using this coding framework to code the first three transcripts in NVivo. Subsequently the codes were discussed with and validated by a second researcher. The codes were then applied to all transcripts, but at the same time additional emerging codes were considered. For document reviews, data was extracted using a data extraction framework, which was developed based on the research questions. Interview data were used to complement, compare, and contrast information from the document review, which allowed for triangulation of findings.

To reduce the influence of background and views of the researchers on data collection, analysis and interpretation, data collection was carried out according to a predefined protocols and pretested tools. Moreover, three researchers were involved in the analysis, which allowed critical discussions and reflexions on concepts, and on interpretation of data, thus addressing potential bias.

### Ethics approval and consent to participate

Ethical approval for this study was sought and obtained from the Ethics Committee of the National Institute of Research in Public Health (N°23/2017/CE-INRSP). Participation was voluntary. Participants were able to stop the interview at any time without explanation. Oral informed consent to participate was obtained from each study participant before each interview. The content of the interview and the identity of the interviewee were kept anonymous.

## Results

The results are presented along the four interrelated components of the Health Policy Triangle.

### Curriculum content

Questions were asked about the content of the harmonised curriculum and the changes and adjustments needed for its adoption. The data from the document review show that the harmonisation policy had a well-defined goal, which was to address disparity in the level of midwifery training in the ECOWAS area, and to align training with the priority health needs in this area. The strategies were to implement a shared harmonised curriculum and to adopt the competency-based approach to teaching.

The programme extends over three full years of training as the objective-based programme. Compared to the objective-based programme, which emphasised the attainment of academic subject content goals, the competency-based approach implied that time investment increased considerably due to the introduction of more practice and internships (see Tables 2 and 3). It also included more clinical skills development related to obstetric emergencies’ management, and to community health and knowledge about the concepts of health research.

**Table 2:** “Classic” objective-based curriculum main components and time investment.

Components	Time (in hours)
Theory	1367
Self-study	0
Internship	712
Practical work in obstetrics	120
Total time investment	2199

**Table 3:** The WAHO competency-based curriculum’s main components and time investment.

Competencies/main topics	Components (time in hours)				
	Theory	Practice	Students’ self-study	Internship	Total
To manage clinical situations by referring to the physiology and diseases of the human body	452	46	336	180	1014
To intervene in community health	100	14	70	45	229
To apply methods and techniques of nursing and midwifery	168	48	96	495	807
To apply the research processes	58	26	64	270	418
To ensure the management of nursing and midwifery care	123	47	96	225	491
To ensure promotional, preventive, and rehabilitative care for the “mother and child”	46	14	40	180	280
To provide high-quality antenatal care for the optimal health of women during pregnancy	60	18	56	90	224
To provide emergency obstetric and neonatal care to maximise the health of mothers and their new-borns	160	56	134	720	1070
To provide culturally acceptable high-quality care during pregnancy, labour, and childbirth.	50	10	40	135	235
Total time investment	1217	279	932	2340	4768

The internship duration has almost tripled (from about 712 hours to 2340 hours), underlining the emphasis on the acquisition of practical competence that was considered insufficient in the objective-based programme. Also, the student’s self-study time that was not officially accounted for before has been considered in the new curriculum for 932 hours. Practical classroom courses increased from 120 to 279 hours. The number of hours allocated to developing each competency is equivalent to the WAHO’s estimation.

From the interviews, factors that influenced the curriculum adoption in schools were related to the relevant stakeholders’ knowledge of the harmonisation policy, as well as their knowledge of the change in the organisation and contents of training. Moreover, the competency-based approach appeared to be a new concept for participants from private schools.

### Actors

Based on the interviews and document review, we identified five groups of actors involved in the policy adoption process (see Table 4). The lack of formal communication and consultation between these different groups of actors, as well as their lack of engagement with policy processes, impinged on its adoption.

**Table 4:** Actors and roles.

	Actors	Roles and influences
Government agencies	Department of Human Resources for Health (DoH)/ Ministry of Health (MoH)	*Employer*. Determines the needs and type of health workers to be recruited for the public sector. Power to act on the recruitment of graduates Power over the health structures to use the graduates
	Department of Public Service (DoPS) / Ministry of public services	*Employer*. Responsible for the recruitment of civil servants. Power to act on the recruitment of graduates
	Department of Local Government (DoLG)	*Employer*. Responsible for the recruitment and employment of civil servants for local government. Power to act on the recruitment of graduates
	Department of Higher Education (DoHE) / Ministry of Higher education (MoHE)	Responsible for regulation of training in higher education in Mali. Power over the training schools to adopt the curriculum
Training schools	Public training school: National Training Institute in Health Sciences (INFSS)	*Public training school*. Responsible for the development of training programmes. Train midwives and nurses Power to act on the adoption of the curriculum
	Private training schools	Train midwives and nurses for the health labour market Power to act on the adoption and implementation of the curriculum
Employers	Health structures etc.	Users of graduates Power to act on recruitment of the graduates
Beneficiaries of the training	Associations and council of midwives and nurses	Responsible for the promotion and/or the regulation of the professions and the practices
	Students	Participation in the training
International agencies	Donors/ Technical partners	Technical and financial support Facilitate adoption by providing technical or financial assistance to institutions and schools.

#### Regulation level

To some extent, all actors have influenced policy adoption. A key stakeholder from the midwives’ association and council expressed support for its adoption, as the new curriculum requires a higher level of education, potentially leading to better pay and increased graduate competency:

*“There is no quality control of the training*. *Everybody does what they want to do*. *The harmonised programme will help to bring some regulation*.*”* [Participant, midwives’ association and council]

According to participants, the MoH, as a member of the ECOWAS Ministries of Health Assembly, played a leading role in the new training programme’s adoption in Mali. However, MoH has made little use of its influence for adoption at the level of training and employment structures, especially at private health training structures. The human resources for health (HRH) department, responsible for staff recruitment and deployment in the public sector, determines the type of graduates to be recruited. The HRH department influenced graduates’ recruitment by deciding not to recruit new graduates in favour of those from the previous programme, who commanded lower salaries. The Department of Public Services, which recruits health workers for the MoH, has the final say in determining the categories of employment in the public services and also determines salary levels. Participants noted that this department opposed the recruitment of the new graduates because they would not fit into the existing salary scheme. The MoHE, which validates higher education programmes in Mali, was not associated with the policy adoption. According to participants, the INFSS was not recognized as a higher education institute and thus did not have to consult the MoHE.

#### Technical level

At the technical level, the INFSS was the first to adopt the new curriculum. Respondents attributed the INFSS’ leading role in the programme’s adoption to its active participation in the programme’s development with the WAHO. However, it had no power over or supervisory responsibility for the other structures in Mali regarding the adoption of the new curriculum.

According to participants from the INFSS, several factors contributed to its success in adopting the programme. Firstly, it was relatively prepared due to its previous experiences with the competency-based curriculum. Secondly, it benefited from donors and international agencies’ support for adoption and implementation. Technical and financial partners (TFP) also played a key role in supporting the government in health workers’ training through training of trainers and providing training materials. The TFP support is a result of the global policy to reduce maternal and child mortality and in efforts to achieve the SDGs. Moreover, the WAHO supported the INFSS through providing support and training for teachers.

From 2017, international support in improving pre-service training was extended to certain private structures. Projects financed by different development partners supported selected schools in terms of teacher training and materials. Support is generally conditional on the adoption of the harmonised curriculum, according to respondents from two TFPs. Despite that, private training schools have the collective power to act on the adoption of the new curriculum. Some have joined forces through a self-regulatory association and determine their own pace in adopting the curriculum:

*“We have instructed all schools affiliated [to the association] to start using the WAHO programme*. *And we organised training sessions for them*. *But we can only speak on behalf of the schools that are members of the association*.*”* [Participant, private training school]

Some key stakeholders–such as professional associations and councils, and students–have not directly been involved in the curriculum adoption. However, they expressed their support for it.

### Process

The interviews and document review explored the process of curriculum adoption, the linkage with the labour market, and the communication about the policy.

#### Curriculum adoption

Findings showed that the adoption of the harmonised curriculum did not follow the process defined by the MoHE to ensure the recognition of diplomas issued by higher education institutions in Mali. According to the regulations, from document analysis and interviews, any new curriculum must have obtained prior approval from the MoHE for diplomas to be acknowledged by public services. This approval will facilitate acceptance of new graduates by the MoH, including the adaptation of salary scales and career paths for midwives. In practice, the documents analysis and interview with the representatives from public schools showed that the curriculum was implemented by the INFSS before its approval by the MoHE. Prior formal approval was not sought from the MoHE because training schools for midwives were not affiliated with the Ministry at the time, and the INFSS was not aware of and did not feel the need to obtain such approval. This is further explained as part of the contextual factors.

The formal approval documents from the MoHE backed to 2015, when 146 graduates (2014 and 2015 graduates) of the new programme were already on the labour market. The late approval constituted a barrier to the recognition of diplomas by the Ministry of Public Service, resulting in unemployment. In addition, this lack of recognition hampered the adoption of the curriculum in private schools, as stated by a participant:

*“We are not using the new curriculum because the Ministry of Public Service is not recruiting them*. *If we adopt the curriculum [while others are not doing so]*, *our graduates would not be recruited*, *and we won’t have new students*.*”* [Participant, private training school]

Interviews with school managers showed the lack of a coordination structure for the curriculum adoption. According to its representative, as the only public training structure for midwives and nurses, the INFSS tried to lead this adoption process, but it only had a collaborative relationship with private schools and no control over or influence on their adoption of the new training curriculum.

Differences in the new curriculum adoption between the INFSS and private schools were observed by participants. As previously highlighted, numerous factors facilitated the INFSS’ adoption of the new curriculum. However, at the private schools, adoption of the new curriculum has been slower and was still not effective in all schools at the time of this study. According to participants, the slowness of adoption may be linked to constraints in the organisation of training. In addition, both of the respondents from private schools viewed the adoption of the new curriculum as a challenge, as the process of change is difficult and resource intensive. They also added that its structure and lack of regulation makes it slow to implement, as illustrated by one of the participants:

*“Schools claim to apply for the WAHO programme*, *but they do not*. *The application of the competency-based approach is very difficult because it requires effort and a lot of materials*. *Teachers must be trained*, *and laboratories equipped*. *Most private schools do not even have permanent teachers and do not have practice rooms or libraries*. *How can the competency-based approach be taken under these conditions*?*”* [Participant, private training school]

*Process-related factors that link the curriculum to the labour market*

The formal lack of approval also affected student recruitment, which ultimately affected private schools’ willingness to implement the new curriculum. Overall, there was no link with the labour market to ensure the integration of new graduates into the health system. From both interviews and document analysis, we observed an absence of a preliminary negotiation process regarding the positions, functions, and salary levels of graduates. A consequence of the lack of discussion to seek consensus on salary scales was that the graduates were not recruited as they are more expensive for employers than those trained under the “classic” (objective-based) curriculum. For the same job, a graduate with a bachelor’s degree using the WAHO curriculum commands twice the salary of a graduate trained through the classic objectives-based curriculum. An HRH manager added:

“*Despite the increase in the budget for the recruitment of health personnel from 600 million CFA francs to more than one billion*, *this is not enough to meet the needs*. *This increase concerns all categories of agents*. *Instead of recruiting 10 agents [with the bachelor’s degree]*, *we can recruit 20 [graduates from the classic curriculum]*.*”* [Manager, HRH department]

#### Communication about the policy

According to graduate employers from private and public training schools who were interviewed, there was a lack of communication between each other. There was no defined dissemination strategy to the different actors prior to adoption of the policy. They also mentioned the weak collaboration of the training schools with employment and recruitment structures. The lack of dissemination contributed to the non-recruitment of graduates by public services because employers were not informed, and the new type of graduates seemed to have come as a surprise to them. According to an INFSS official:

“*The Ministry of Public Service has refused to recruit graduates because*, *they said*, *they do not recognize the new graduates despite the technical note we sent them to explain the programme harmonisation process in the region and the fact that all ECOWAS countries had to adopt it*.*”* [Participant, public training school]

### Context

From interviews and the document review, contextual factors that influenced the adoption of the harmonised training curriculum in Mali were the reform of higher education in Mali and in West Africa, the human resources for maternal health situation and HRH policy in Mali, and the regulation of training schools and accreditation of training programmes.

#### Reform in higher education system in Mali and West Africa

In 2008, Mali engaged in a reform of its higher educational system with the adoption of the Bachelor (Licence)-Master–Doctorate (LMD) system. The reform followed the West African Economic and Monetary Union (WAEMU) 2007 directive, inviting its member states to adopt the system by 2011, with the objective of improving the relevance and quality of higher education and research in the West African region. This directive is like that of the African and Malagasy Council for Higher Education, which acts as a supranational body for the approval and accreditation of diplomas. The Council’s resolution aims to build: “an open and harmonised African and Malagasy higher education area.”

These resolutions clearly refer to a reform in education known as the Bologna process, which aimed to create convergence in higher education among several European countries with an educational structure at the LMD level. The Bologna process came to Mali in 2008 through a presidential decree requiring all higher education institutions in the country to conform to the LMD system by 2012. The reform is of great importance to educational programmes for nurses and midwives, raising their educational status from a diploma to a graduate level. However, it must be noted that while the objective-based curriculum has been de facto considered to be at the LMD level by training schools, its graduates are not considered as holding a bachelor’s degree at recruitment.

It is in these contexts that WAHO approached the harmonisation of training programmes at the sub-regional level, going hand in hand with the LMD system. The finalisation of the WAHO’s harmonised training curriculum for midwives in 2012 and its approval by the ECOWAS Assembly of Ministers coincided with the above-mentioned transitions in higher education in Mali. This occurrence offered an opportunity to adopt the WAHO curriculum, at least at the INFSS. An INFSS participant, involved in its management at the time of transition, stated a reason for its willingness to adopt the new curriculum:

“*INFSS had been seeking recognition as a higher education institution and a recognition for their training staff for several years without success*. *The adoption of the new curricula and the LMD system was a facilitator for this to happen*.” [Participant, public training school]

### HRH in Mali

The health system in Mali faces a significant deficit in midwives, with less than one midwife per 5,000 persons. According to human resources managers, those deficits have been partly resolved by opening training to private structures. The deficits have also been aided by raising the level of training from a junior high school diploma to a baccalaureate in 1997 with the objective-based programme. However, the quality of graduates is still considered inadequate by employers due to a lack of training regulation and outdated curricula.

### HRH policy

According to HRH managers, a major constraint on Mali’s health system includes issues in human resources, which need to be prioritised to achieve health objectives. To address these issues, a five-year human resources policy was developed in 2009. This policy aimed at increasing the production capacities of training schools, improving the quality, recruitment, motivation, and career development for health workers (including midwives), among other benefits. However, according to the interviewee at the HRH directorate, it has not been implemented due to associated costs. Since then, no other plan has been developed, which implies that the harmonisation policy is not embedded in any HRH plan. This hampers its integration in HRH systems, such as adapted cadres and salary scales.

#### Regulation of training schools and accreditation of training programs

According to interview participants, the difficulty in implementing the harmonised programme is linked in part to the multitude of training schools and their lack of regulation. From the document analysis and from interview data, there is no functional accreditation system in place for midwives’ and nurses’ training in Mali. In 2016, the supervision of training institutions for health professionals was transferred from the MoH to the MoHE, which has few effective coordination mechanisms. This means that only a small number of private training schools have had their training programme validated by the MoHE, according to a respondent from the Ministry. There is no accountability mechanism in place. According to a participant:

*“The problem with [midwifery] training now is the plethora of training schools*. *There is no control*, *schools open every day*. *There is no seriousness in private schools*. *People do as they please*.*”* [Participant, Association and council]

According to a representative of the association of private schools, schools often open without having all the necessary authorisation. Some of the private schools have formed an association to self-regulate their practice, but most schools are independent and not affiliated to this association.

## Discussion

Studies analysing the adoption of policies, such as training policies, are scarce in low-income countries [27]. While the harmonisation policy was aligned with Mali’s health goals, it faced a number of barriers in its adoption. The most salient factors that influenced adoption were: 1) (lack of) available resources, and 2) (lack of) relevant relations and networks necessary for successful adoption, which pertained to the (lack of) involvement of relevant actors and (lack of) coordination and communication between actors.

### Resources

One of the most common issues influencing the adoption of health innovations in LMICs is access to financial resources [28]. Barriers to adoption include inadequate distribution of resources and a lack of local, sustainable funding sources [28]. Health workforce scale-up and improvement require substantial financial resources as they often necessitate initial training costs, as well as investments and recurring costs for upscaling HR capacity [29]. While the WAHO is instrumental in facilitating the attainment of regional health goals through the harmonisation of health policies, for example, it has little funds to ensure their implementation. Consequently, each country must strategically plan how to include the initiative in its list of priorities within a resource-constrained context. Countries are likely to have to rely on international donors for funding, which does not guarantee (sustainable) prioritisation of the WAHO’s initiatives. For example, recent efforts to introduce a new HRH program in Rwanda were met with challenges stemming from year-by-year donor commitment renewals, withdrawal of donor commitment, as well as spending restrictions on crucial items like monitoring and evaluation [30]. The INFSS was able to adopt the new curriculum as they received additional resources such as training and other technical support, predominantly offered by international donor organisations. However, a driving factor behind the lack of adoption of the new curriculum in both private schools included in the study was the lack of access to such resources. Therefore, the influence of donor aid in successful adoption demonstrates a major barrier imposed by the lack of local funding sources in Mali.

While the reliance on donor aid can be challenging and unreliable, as demonstrated by the program in Rwanda, there are ways to counterbalance it. One way is increasing local ownership. This could be achieved by funnelling donor funds directly to the MoHE instead of to individual training institutions or through intermediaries [30]. Subsequently, the MoHE could utilise the funds in a consistent manner to support a regulatory board for supervising the training of all healthcare professionals such as through management, human resources, and coordination training. Another important resource would be for the MoHE to provide support to individual schools on how to apply for funding through capacity building, so as to diversity funds and to ensure that each school can access the necessary resources for adopting the new curriculum. This capacity building along with decentralisation of fund-raising to individual schools would lead to increased financial sustainability. An innovative method used to increase such an approach’s sustainability includes a “reverse funds flow” model, where the program’s managing body (or the MoH/MoE) uses donor funds to contract peers from HIC to provide scientific and technical consultation [31]. Once initial training has occurred, local individuals could be contracted to provide training and further technical support—for example, teachers trained to provide the new curriculum could be required to devote time to support others [31].

At the regulatory level, policy adoption was hampered by resource constraints imposed by the introduction of a new category of personnel and salary requirements. Paradoxically, Mali is experiencing acute underemployment of healthcare workers, yet is simultaneously investing in producing more healthcare workers [32]. Findings exemplified that in addition to this phenomenon, many of the newly harmonised curriculum trained midwives require higher salaries than others, resulting in even fewer opportunities for employment. As a result, it is evident a mismatch exists between the supply and demand of health workers, and that the process of adopting the harmonisation policy was fragmented and inadequate. A more appropriate adoption process would have been aided by first conducting a health labour market analysis to achieve a better understanding of key issues in Mali’s health system prior to policy adoption [32]. Such a study would ideally establish relationships between policymakers and local research partners, who could provide feasibility analyses of proposed policy recommendations. A stakeholder analysis and contextual information that could influence decision-making would also be a relevant aspect of the analysis [32]. Integrating such information into policy development would ensure a more considered and feasible adoption strategy. However, it appears that the adoption strategy in Mali failed to consider the labour market implications, where those responsible for hiring new graduates had no negotiating and decision-making power or role in integrating the new midwife category in Mali’s health system’s organisational structure prior to policy adoption. Consequently, this has significant implications for new graduates and proves to be a major barrier to improving the quality of maternal care through a strategy of upgrading pre-service training.

Additionally, the ongoing conflict in Mali is likely to have a substantial impact on efforts for educational reform. In recent years, the political and security context has been particularly unstable. Its territorial integrity was seriously threatened in 2012 with the occupation of parts of the country and a coup that triggered a political crisis. The political insecurity has since moved to the centre of the country. Subsequently, the health system has been impacted by the reallocation of state resources to priority security issues, which may have affected not only the recruitment of graduates, but also halted any other efforts to improve training. This effect has also been exacerbated by strikes for salary increases amongst multiple professions across the country, which might challenge any reform that would place additional pressure on the salary bill. As a result, this aspect of Mali’s context poses a major barrier to the adequate distribution of resources required for successful adoption of the harmonised curriculum.

### Collaboration and coordination between relevant actors

While contextual factors hindered policy adoption, there were additional factors that also played a substantial role. A major barrier to policy adoption is weak regulatory enforcement, which is related to the lack of involvement and support of relevant actors [28]. Findings support this statement, as it was clear that harmonisation efforts in Mali did not include all relevant stakeholders. The decision to adopt the harmonised midwifery curriculum was essentially a decision taken between the MoH cabinet and the INFSS, without reference to other actors both inside and outside of the health and training systems. Policy adoption was not part of a process that brought together relevant actors to formulate needs and define the adoption and implementation strategies for this new policy, including the integration of new graduates into the organisational structure, which ultimately impacted its success in practice.

The weak regulatory enforcement alludes to several system flaws, such as inadequate dissemination methods, inadequate leadership, and a lack of integrated and coordinated relationships between organisations in the health system [33]. In line with results from studies in other LMICs [34, 35], findings from this study were similar. Our results demonstrate a lack of defined communication strategies and a lack of coordination between the different structures in charge of adoption. Ultimately, the necessary relations and networks for the policy adoption’s success were not in place.

Health workforce development is a technical and political process that depends on different sectors and groups in society and different levels of government, which subsequently requires adequate coordination and establishment of partnerships [36–39]. For example, decisions that influence the health labour market, such as education policies and remuneration, reach beyond the technical and sectoral remit of health ministries. Alongside policy formulation, adoption allows for the creation of alternatives, deliberation, advocacy, lobbying, negotiation, and guidance for implementation [40]. A limiting factor in the policy adoption in Mali was the lack of involvement of the actors responsible for post-training management, such as graduate employment. Relevant actors for collaboration include other government ministries like labour and finance, as well as regulatory bodies, the private schools, and international development partners. Importantly, they should be consulted early in the policy process to ensure meaningful involvement and optimal success [32, 41]. This should include regular meetings with decision-makers to increase their trust in and ownership over the policy. A robust coordination process, including ownership by different stakeholders, would likely have had a positive influence on policy adoption.

### Strengths and limitations

A limitation of the study is the limited number of participants from the private sector, as well as from outside of urban settings due to security issues to meet people outside the capital. This may not have allowed these stakeholders’ perspectives to be fully explored.

A strength of the study is the diversity of stakeholders included that allowed us to gain insight into the adoption process from different perspectives. It is also one of the few studies on this problem in LMIC. Moreover, the results are generalizable to other countries with similar contexts in the region engaged in training reform. Further studies should explore the implementation process and the outcomes of the programme.

## Conclusion

This study examines the adoption of the WAHO’s harmonised curriculum in Mali which was intended to contribute to the quality of maternal and child health. Commitment of the public school and the MoH and the political context were central to achievements. However, factors linked to the availability and distribution of resources and the lack of involvement of, and coordination between, relevant actors impeded the curriculum’s successful adoption, as well as graduate employment rates in the public sector.

## Supporting information

S1 File Interview guide French. (DOCX)

S2 File Interview guide English. (DOCX)

S3 File Reviewed documents list. (DOCX)
