# Supplementary material for: Adoption of harmonisation policy for the midwives’ training programme in Mali: A policy analysis
Source: PLOS Glob Public Health. 2022 Nov 29;2(11):e0001296. doi: 10.1371/journal.pgph.0001296 (PMC10022231; doi:10.1371/journal.pgph.0001296)
Supplement: S1 File — (DOCX) [file pgph.0001296.s001.docx]

**Guide d’entretiens sur la politique de formation des sages femmes.**

*Destinataires : décideurs politiques, informateurs clés*

1. Politiques en matière de ressources humaines pour la santé.
   1. Sur le **développement et la production** des ressources humaines :
      1. Quelle est l’orientation voulue (au niveau central), pour le développement des ressources humaines en santé ?
      2. Est-ce qu’il y a des objectifs chiffrés par année ?
      3. Comment sont évalués les besoins en ressources humaines et comment sont-ils pris en compte ?
      4. Quelle est la régulation entre les formations publiques et privées ?
      5. Qui gère cette régulation ?
      6. Pouvez-vous décrire la nouvelle politique relative à la formation des sages-femmes au niveau licence ?
   2. Sur le **recrutement** des ressources humaines :
      1. Comment sont évalués les besoins en recrutement et par qui ?
      2. Qui décide du recrutement des différents personnels de santé ? (Quelle est l’implication de la Direction des Ressources Humaines ?)
      3. Quel est le processus de recrutement, quelles en sont les étapes ?
      4. En raison des différentes sources de recrutement, il y a de grandes différences de statut et de traitement entre les différents membres des centres de santé, ce qui pose des problèmes. Est-ce un problème qui est pris en compte, par qui est-ce géré ?
2. Politiques spécifiques aux sage-femmes
   1. Reposer les mêmes questions (développement et production).
   2. Questionner également la chronologie des politiques mises en œuvre :
      1. Qu’en est-il aujourd’hui ?
      2. Lesquelles de ces politiques ont eu des impacts, surtout en milieu rural ?
      3. Ces impacts ont-ils été mesurés, comment ?
   3. Pourquoi la stratégie de formation des sage-femmes au niveau licence a-t-elle été mise en place ? (*Une évaluation préalable de la compétence des SF a-t-elle été faite, quel était l'objectif de cette stratégie*) ?
   4. Par qui cette stratégie a-t-elle été élaborée ?
   5. Qui ont été les exécutants de cette stratégie ?
   6. Comment la nouvelle formation au niveau licence a-t-elle été adoptée et mise en œuvre dans les écoles ?
   7. Qui sont les personnes impliquées dans l'adoption, la mise en œuvre du programme et la formation ?
   8. Quelles sont les écoles qui peuvent offrir cette formation ? Pourquoi ? Comment ?
   9. Comment le niveau de formation et d'examen est-il réglementé dans les écoles publiques et privées ?
   10. Comment a évolué le recrutement des sages-femmes par l'Etat au cours des 5 dernières années, avant et après l'augmentation du niveau de formation ?
   11. Quelle est la vision du gouvernement pour l'évolution future du recrutement en matière de santé sexuelle, reproductive, maternelle et néonatale (NSMS) ?
