# Supplementary material for: Adoption of harmonisation policy for the midwives’ training programme in Mali: A policy analysis
Source: PLOS Glob Public Health. 2022 Nov 29;2(11):e0001296. doi: 10.1371/journal.pgph.0001296 (PMC10022231; doi:10.1371/journal.pgph.0001296)
Supplement: S2 File — (DOCX) [file pgph.0001296.s002.docx]

**Interview guide on midwifery education policy.**

*Target audience: policy makers, key informants*

1. Human resources for health policies.
   1. On human resource **development and production**:
      1. What is the desired direction (at central level) for health human resource development?
      2. Are there numerical targets per year?
      3. How are human resource needs assessed and how are they considered?
      4. What is the regulation between public and private training?
      5. Who manages this regulation?
      6. vi. Can you describe the new policy on midwives training?
   2. On human resources **recruitment:**
      1. How are recruitment needs assessed and by whom?
      2. Who decides on the recruitment of different health personnel? (What is the involvement of the Human Resources for Health Department?)
      3. What is the recruitment process, what are the steps?
      4. Due to the different sources of recruitment, there are big differences in status and treatment between different members of the health centres, which causes problems. Is this a problem that is being addressed, by whom is it being addressed?

1. Specific policies for midwives
   1. Ask the same questions again (development and production).
   2. Also question the chronology of the policies implemented:
      1. What is the situation today?
      2. Which of these policies have had impacts?
      3. Have these impacts been measured and how?
   3. Why was the strategy of educating midwives at baccalaureate degree put in place (*was there a prior assessment of the competence of FS, what was the purpose of this strategy*)?
   4. By whom was this strategy developed?
   5. Who were the executors of this strategy?
   6. How has the new training been adopted and implemented in schools?
   7. Who are the people involved in adoption, programme implementation and training?
   8. Which schools can offer this training? Why? How?
   9. How is the level of training and examination regulated in public and public schools?
   10. How has the recruitment of midwives by the government evolved over the last 5 years, before and after the increase in the level of training?
   11. What is the government's vision for the future development of sexual, reproductive, maternal, and newborn health (SRNH) personnel recruitment?
