# Supplementary material for: Adoption of harmonisation policy for the midwives’ training programme in Mali: A policy analysis
Source: PLOS Glob Public Health. 2022 Nov 29;2(11):e0001296. doi: 10.1371/journal.pgph.0001296 (PMC10022231; doi:10.1371/journal.pgph.0001296)
Supplement: S3 File — (DOCX) [file pgph.0001296.s003.docx]

List of reviewed documents.

1. WAHO. Strategic Plan 2016-2020. West African Health Organization; 2016.
2. WAHO. Harmonized training curriculum for nurses and midwives in ECOWAS region/. West African Health Organization; 2014.
3. Direction générale de l’enseignement supérieur et de la recherche scientifique. Rapport d’activités 2019. Bamako, 2020.
4. Décret n°06-395/P-RM du 19 septembre 2006 fixant les modalités de l’habilitation et de la délivrance des diplômes de l’Enseignement supérieur. Mali2006.
5. INFSS. Rapport d’activités 2014. Institut National de Formation en Sciences de la Santé ; 2015.
6. INFSS. Rapport d’Activités 2015. Institut National de Formation en Sciences de la Santé ; 2016.
7. Directive n°03/2007/cm/uemoa portant adoption du système licence, Master, Doctorat (LMD) dans les universités et établissements d’enseignement supérieur au sein de l’UEMOA (2007).
8. Décret N°08-790/P-RM du 31 Décembre 2008 portant institution du système Licence-Master-Doctorat (LMD) dans l’enseignement supérieur au Mali, (2008).
9. General Convention A/C/1/01/03 on the Recognition and Equivalent of Degrees, Diplomas, Certificates and Other Qualifications in ECOWAS Member States, (2003).
10. Protocol A / P.3 / 01/03 on Education and Training in ECOWAS region, (2003).
11. DRH. Profil pays des ressources humaines en santé du Mali 2016. Direction des ressources humaines secteur santé et développement social, ministère de la Santé et de l’hygiène publique ; 2017.
12. Loi n°99-046 du 28 décembre 1999 portant loi d’orientation sur l’éducation
13. Ordonnance N°04-032/P-RM du 23 septembre 2004 portant création de l’Institut National de Formation en Sciences de la Santé
14. Arrêté N°2012-1915/MESRS-SG du 11 juillet 2012 Portant création et Organisation du diplôme de Licence Professionnelle dans les structures d’enseignement supérieur en République du Mali.
15. Arrêté N°2012-1916/MESRS-SG du 11 juillet 2012 Portant Organisation du diplôme de Licence Professionnelle dans les structures d’enseignement supérieur en République du Mali.
16. Arrêté N°2012-1915/MESRS-SG du 11 juillet 2012 Portant création et Organisation du diplôme de Master dans les structures d’enseignement supérieur en République du Mali.
17. Décret N°04-466/P-RM du 20 octobre 2004 Fixant l’organisation et les modalités de fonctionnement de l’Institut National de Formation en Sciences de la santé.
18. Evaluation à mi-parcours du Programme de Développement Socio-Sanitaire (2014-2018) PRODESS III Rapport final
19. OMS, UNFPA, ministère de la Santé. Rapport d’évaluation de la qualité de la formation initiale des sages-femmes au Mali. 2016.
20. Cellule de planification et de statistique secteur sante, développement social et promotion de la famille. Synthèse des rapports d’activités 2017 et de programmation 2019 pour le comité de suivi du programme de développement sanitaire et social. 2018.
21. Cellule de planification et de statistique secteur santé développement social et promotion de la famille. Programme de développement socio-sanitaire 2020-2023 (PRODESS IV).
22. Cellule de planification et de statistique secteur santé développement social et promotion de la famille. Programme de développement socio-sanitaire 2014-2018 (PRODESS III).
23. DGESRS. Créer et ouvrir un établissement privé d’enseignement supérieur n.d. <https://dg-enseignementsup.ml/creer-et-ouvrir-un-etablissement-prive-denseignement-superieur/>
24. Arrêté N°2021-3206/MESRS-SG du 25 Août 2021 Fixant la liste des programmes de Formation Habilités d’institution privées d’enseignement supérieur.
25. Arrêté N°2017-0344/MESRS-SG du 16 fev2017 Fixant la liste des programmes de Formation Habilités d’institution d’enseignement supérieur.
